# Introduction of Medicinal Plants Species with the Most Traditional Usage in Alamut Region

**Published:** 2012

**Authors:** Maryam Ahvazi, Farahnaz Khalighi-Sigaroodi, Mohammad Mahdi Charkhchiyan, Faraz Mojab, Vali-Allah Mozaffarian, Hamideh Zakeri

**Affiliations:** a *Department of Herbarium, Institute of Medicinal Plants, ACECR, Karaj, Iran.*; b *Department of Pharmacognosy and Pharmaceutics, Institute of Medicinal Plants, ACECR, Karaj, Iran *; c *Research Institute of Forests and Rangeland, Ghazvin.*; d *School of Pharmacy and Pharmaceutical Sciences Research Center, Shahid Beheshty University of Medical Sciences, Tehran, Iran.*; e *Research Institute of Forests and Rangeland, Tehran, Iran.*; f *Cell Line Engineering, Sigma Aldrich Biotechnology Division. St Louis, USA.*

**Keywords:** Medicinal plants, Ethnobotany, Alamut, Ghazvin, Iran

## Abstract

The ethnobotany of the medicinal plants of Alamut region is important in understanding the cultures and traditions of Alamut people. This study documents 16 medicinal plant species, most commonly used by the indigenous people of Alamut region (Ghazvin Province), northwest, Iran. The botanical name, family name, vernacular name, part used, and the application of the plants have been provided in this paper. Alamut region was divided into different villages with the aid of maps. We recorded traditional knowledge and use of medicinal plants from herbal practitioners and village seniors in Alamut. The plants were gathered from different sites. The fully dried specimens were then mounted on herbarium sheets. We found 16 medicinal plants belonging to 11 families which were traditionally used in Alamut. Finally, we describe traditional usages by the native people in the Alamut region. The obtained results were compared with data on the herb’s clinical effects. A set of voucher specimens were deposited to the Institute of Medicinal Plants Herbarium (IMPH).

## Introduction

Before the introduction of chemical medicines, man relied on the healing properties of medicinal plants. Some people value these plants due to the ancient belief which says plants are created to supply man with food, medical treatment, and other effects. It is thought that about 80% of the 5.2 billion people of the world live in the less developed countries and the World Health Organization estimates that about 80% of these people rely almost exclusively on traditional medicine for their primary healthcare needs. Medicinal plants are the “backbone” of traditional medicine, which means more than 3.3 billion people in the less developed countries utilize medicinal plants on a regular basis (1). There are nearly 2000 ethnic groups in the world, and almost every group has its own traditional medical knowledge and experiences (2, 3). Iran is home to several indigenous tribes with a rich heritage of knowledge on the uses of medicinal plants. Iran has varied climates and geographical regions that have caused a wide distribution of individual medicinal plant species such that each tribe has its own plants and customs. Alamut is one of the most important geographic regions in Iran because of its ancient history of cultivating traditional medicinal plants. Alamut region and the several villages it encompasses are secluded from other cities in Iran, which is why the people living in this region have relied on indigenous medical knowledge and medicinal plants. In this study, we analyzed the medicinal plants with most therapeutic usage in the region.

## Experimental

*Geographic and climatic overview*

**Figure 1:**
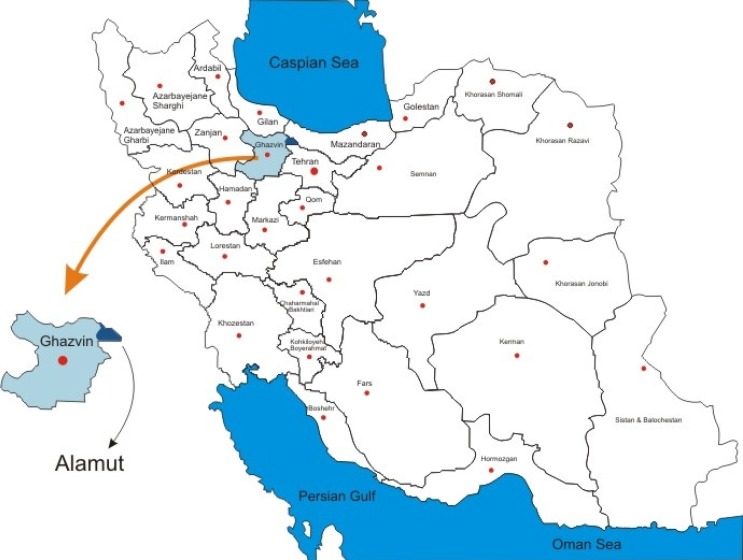
Study area: Iran map and Alamut in Ghazvin Province

Alamut mountainous region is situated in the central Alborz Mountains, between 36˚24´ and 36˚46´ northern latitudes and 50˚30´ and 50˚51´ eastern longitudes with an altitude ranging from 2140 to 4175 m. The region is located on the northeast of Ghazvin Province and is bounded to the north by the Mazandaran Province in Tonekabon and bounded on the east by Tehran Province in the Taleghan mountains. Annually, it rains 368.03 mm and the average temperature is 14°C. Topography is distinctly marked with several mountains, springs, rivulets, and rivers. This area is geographically located in the Irano-Turanian region (Figure 1).

The ethnic composition of the region is quite diverse and almost 90% of its population resides in rural areas. The language of the inhabitants is known as Deylamite. People of Alamut have a long history of exporting medicinal plants to other regions of Iran. Roadways have increased communication among the rural natives in Alamut and have also increased tourism to the region because of its several ancient castles. Because of good quality of medicinal plants in this region and more immethodical pick of them, some of species have become extinct. For this reason, an important aim of this study is to protect the preservation of the region’s plants. Other aims include:

Documenting the traditional knowledge of medicinal plants from the natives.

Assessing the most commonly used local medicinal plants.

Promoting the potential benefits of medicinal plants.

*Data collection*

We first prepared a map with a scale of 1:25,000 from the region to identify the number of villages, roads, and vegetations. We visited the region and spoke to herbal practitioners and village seniors. A questionnaire was used to obtain information on the types of ailments treated using traditional medicinal plant species. Sometimes informants were asked to come to the field and introduce us to the plants. When this was not possible, plants were collected around the villages of the informants and were shown to them to confirm the plant names. This investigation took over 2 years and information was collected 1-2 days per week. Voucher samples were also collected for each plant and were identified using floristic, taxonomic references. Flora Iranica and a dictionary of Iranian plant names were used for identification purposes (4, 5). Plants were deposited at the herbarium of Institute of Medicinal Plants (IMPH).

## Results

Although ancient sages through trial and error methods have developed herbal medicines, the reported uses of plant species do not certify their efficacy (6). Reports on ethnomedicinal uses of plant species require pharmacological screenings, chemical analyses, and tests for their bioactive activities. Pharmacological screening of plant extracts provides insight to both their therapeutic and toxic properties as well as helps in eliminating the medicinal plants or practices that may be harmful (7).

This study provides information on 16 medicinal plants belonging to 12 families that are most commonly used for traditional medicine in Alamut region. Botanical names of plants were sorted alphabetically, and for each species and the following information was hence represented: family, vernacular name, part used (Table 1). Traditional use and preparation was compared with other references (Table 2). 

**Table 1:** Medicinal plants collected from Alamut region

**Botanical name/Voucher specimen no.**	**Family**	**Vernacular name**	**Part used**
*Achillea millefolium * **L. Ahvazi 422 (IMPH)**	Asteraceae	Boomadaran	Aerial parts
*Anchusa italica * **Retz. Ahvazi 457 (IMPH)**	Boraginaceae	Gav zaban	Flowers
*Berberis integerrima * **Bge. Ahvazi 455 (IMPH)**	Berberidaceae	Vialesk	Fruits, roots
*Capparis spinosa * **L. Ahvazi 636 (IMPH)**	Capaeidaceae	Kabar gol, kafar gol	Fruits, blooms, roots without bulk, flowers
*Echium amoenum * **Fisch and May. Ahvazi 637 (IMPH)**	Boraginaceae	Gole gavzaban	Flowers
*Ferula persica * **Willd. Ahvazi 407 (IMPH)**	Apiaceae	Jarand	Stems, roots, leaves
*Fumaria asepala * **Boiss. Ahvazi 638 (IMPH)**	Fumariaceae	Shatare	Aerial parts
***Botanical name/Voucher specimen no.***	**Family**	**Vernacular name**	**Part used**
*Grammosciadium platycarpum * ***Boiss.and Hausskn. Ahvazi 409 (IMPH)***	Apiaceae	Jafari kohi	Leaves
*Heracleum persicum * ***Desf. ex Fischer Ahvazi 410 (IMPH)***	Apiaceae	Golpar	Flowers, seeds
*Hippophae rhamnoides * ***L. Ahvazi 494 (IMPH)***	Elagnaceae	Kaham	Fruits
*Juglans regia* *** L. Ahvazi 639 (IMPH)***	Juglandaceae	Gerdo	Leaves, fruits, fresh aerial parts
*Malva neglecta * ***Wallr. Ahvazi 541 (IMPH)***	Malvaceae	Paniraki, pandiraki	Flower, leaves, roots
*Smyrnium cordifolium * ***Boiss. Ahvazi 640 (IMPH)***	Apiaceae	Avandol	Stems
*Stachys lavandulifolia * ***Vahl Ahvazi 534 (IMPH)***	Lamiaceae	Kaklikoti	Flowers
*Viola odorata * ***L. Ahvazi 593 (IMPH)***	Violaceae	Banafshe	Flowers
*Ziziphora clinopodioides * ***Lam. Ahvazi 538 (IMPH)***	Lamiaceae	Kakoti	Aerial parts
*Ziziphora clinopodioides * ***Lam. Ahvazi 538 (IMPH)***	Lamiaceae	Kakoti	Aerial parts

**Table 2:** Comparison of problems due to hot flash in studied groups during the study base on HFQ.

**Botanical name**	**Traditional Preparation**	**Traditional uses in Alamut region**	**Traditional and medicinal uses in references**
*Achillea millefolium*	Infusion, decoction	Backache, asthma, pectoralgia, infections of pregnant women	Tonic, antihemorrhoids, healing the wounds (8), diaphoretic, emmenagogue (9), cholagogue, antibacterial, astringent, loss of appetite, dyspeptic complanints, liver and gallbladder complaints (10), anti-inflammatory, antispasmodic (used in cold, flatulent colic, heartburn), cicatrizant, antidysenteric, antihaemorrhagic, antipyretic, diuretic, urinary antiseptic (11) to heal chapped skin, haemostatic, hair tonic, decrease blood pressure, antispasmodic, flatulence (11, 12)
*Anchusa italica*	Infusion, decoction	Common cold	Stimulant, tonic, demulcent; used in bilious complaints, fever, cough, asthma; as diuretic in bladder and kidney stones (11), sedative (8, 13)
*Berberis integerrima*	Infusion, decoction, edible	Enteric fever, hyperlipidemia, diabetes, anemia	Enhances the antibacterial activity of ampicillin against *Staphylococcus aureus *(14), antibilious (13), hepatoprotective activity (15), control of hypertension as ACEIs (16)
*Capparis spinosa*	Edible, decoction and demulcent of root	Headache, renal calculus, pododynia, Blooms after boiling are used in some foods because of its hot effects	Diuretic, tonic, antihysteria, gout (17), astringent, diuretic, expectorant, stimulating tonic, gastrointestinal infections, diarrhea, rheumatism, eye infections (18), carminative, headache, blood fat and sugar, hemorrhoids, digestive disorders (19), antibacterial and antifungal activity (20), anti-inflammatory, deobstruent to liver and spleen, anthelmintic, vasoconstrictive (11). Bark: given in splenic, renal and hepatic complaints (11). Juice of leaves and fruits: anticystic, bactericidal and fungicidal (11). Dried flower buds: used in scurvy (11), spleenomegaly, vomiting (21)
*Echium amoenum*	Infusion	Common cold, stomachache, headache, sedative	Common cold, sedative, exhilarating, diuretic (22), analgesic (23), antioxidant, anxiolytic (24, 25), diaphoretic (8)
*Ferula persica*	Steam cooked, edible	Spicy, cooking, heart oxygenating, gout, sinusitis, pododynia, backache	Carminative, diuretic, laxative, alexipharmic, digestive, emmenagogue, antispasmodic (26), hot and dry effects, anti-flatulence, renal calculus, arthralgia, gout, stomach worms, diuretic (27), antihysteria (28)
*Fumaria asepala*	Powder with henna	Migraine, hand schism, mange	Sedative, diuretic, hypotensive and weight reducing (29)
*Grammosciadium platycarpum*	Edible	Tonic, cooking some foods	Antibacterial (30, 31)
*Heracleum persicum*	Infusion, powder, decoction	Tremor, migraine, headache caused by sinusitis (It is harmful for eyes), ascaris worms	Spice, flatulence, indigestion,(28), anticonvulsant activity (32), anti-inflammatory and analgesic properties (33)
***Botanical name***	**Traditional Preparation**	**Traditional uses in Alamut region**	**Traditional and medicinal uses in references**
*Hippophae rhamnoides*	Infusion, edible	Hypertension, hyperlipidemia	Vitamin C content, wounds, epithelization, sclerosis, infection prophylaxis, radiation damage, such as X-ray damage, sunburn, treatment of wounds (10), antioxidant activities (34), prevention of ethanol-induced ulcer formation in rats (35), cancer therapy, cardiovascular diseases, treatment of gastrointestinal ulcers, skin disorder and as a liver protective agent (36), antiworm, fruits are laxative (37)
*Juglans regia*	infusion	Diabetes, backache, pododynia, gonalgia	Skin-excessive, inflammation skin, gastrointestinal catarrh, anthelmintic (10), asthma and sexual weakness (38), psycoanaleptic (39), diabetes (37)
*Malva neglecta*	Edible, Infusion	Constipation, infected boils, mouth fungal infection in children	Lenient, sedative, diuretic, pectoralgia, anti-inflammatory, hemorrhoid, ophthalmitis, vaginite anti-inflammatory, aphtes (17), to heal abdominal pains (40)
*Smyrnium cordifolium*	Edible	Bitter aromatic, hot effects, cooking, tonic	Antimicrobial activity (41,42) edible, diuretic, tonic, removing renal calculus (43)
*Stachys lavandulifolia*	Infusion, powder, edible	Headache, renal calculus	Strengthening stomach, stomachalgia, sedative, digestion tract problems (13), anxiolytic effects (44, 45)
*Viola odorata*	Infusion	Decrease blood pressure, fever, migraine, sedative, constipation	Chronic bronchial asthma, cold, symptoms of the upper respiratory tract, catarrh, rheumatism, skin diseases, inflammation of the oral mucosa, nervous strain, headache, insomnia, hysteria (10), diaphoretic (17), antipyretic (46)
*Ziziphora clinopodioides*	Infusion, edible	Cold, infections, stomachache, headache, increase nausea	Strengthening stomach, stomachalgia, typhus, cold, antiseptic (37,13), antibacterial activity (47), supported stomach, heart ailment (37)
*Stachys lavandulifolia*	Infusion, powder, edible	Headache, renal calculus	Strengthening stomach, stomachalgia, sedative, digestion tract problems (13), anxiolytic effects (44, 45)
*Viola odorata*	Infusion	Decrease blood pressure, fever, migraine, sedative, constipation	Chronic bronchial asthma, cold, symptoms of the upper respiratory tract, catarrh, rheumatism, skin diseases, inflammation of the oral mucosa, nervous strain, headache, insomnia, hysteria (10), diaphoretic (17), antipyretic (46)
*Ziziphora clinopodioides*	Infusion, edible	Cold, infections, stomachache, headache, increase nausea	Strengthening stomach, stomachalgia, typhus, cold, antiseptic (37,13), antibacterial activity (47), supported stomach, heart ailment (37)

Among these medicinal plants, *Apiaceae, Lamiaceae*, and *Boraginaceae *were the most dominant families with 4, 2, 2 species belonging to 4, 2, 2 genera of medicinal plants, respectively.

Of the 16 medicinal plants, 8 species had similar effects in traditional and medicinal uses when comparing Alamut with other references. *Achillea millefolium *had antibacterial effects; *Capparis spinosa *is used for headache, renal complaints and stimulating tonic; *Echium amoenum *is used for common cold and had sedative effects; *Ferula persica *is used for gout; *Juglans regia *is used for diabetes; *Smyrnium cordifolium *is edible and used as tonic; *Viola odorata *is used for fever and migraine; *Ziziphora clinopodioides *is used for cold, infections and stomachache. 

Some effects which are mentioned in traditional medicine of Alamut region were important with no scientific information about them. For example, *Berberis integerrima *and *Hippophae rhamnoides *had good effect on lowering of serum lipids and blood sugar and hypertension. *Malva neglecta *is used for mouth fungal infection in children and *Stachys lavandulifolia *is used for headache and renal calculus. Other researches can perform experiments to discover their components and effects.

All of the medicinal plants were collected from the wild or in the native people’s gardens. Some medicinal plants can no longer be found in the region and are only cultivated in the native people’s gardens. For example, *Echium amoenum *is an endemic plants in Iran with historically wide spread in the region, but because of frequent picking, the species is now just cultivated in the native people’s gardens.

**Figure 2:**
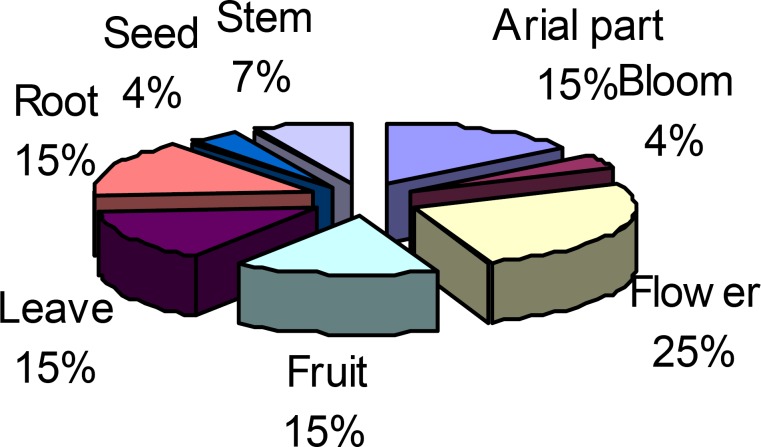
Plants part use and their percentage

Different parts of medicinal plants were used by the inhabitants of Alamut region as medicine for treating ailments. The most common parts used were flowers (25%). The use of aerial parts, leaves, fruits and roots were the same (15%). Use of the stems (7%), seeds, and blooms (4%) were lower than the others (Figure 2). The 16 medicinal plant species were used in treating 27 different types of ailment (Table 3).

**Table 3:** Medicinal plant species were used in treating different types of ailment

No.	Ailment	*Medicinal plants*
1	Anemia	*Berberis integerrima*
2	Ascaris worms	*Heracleum persicum*
3	Asthma	*Achilla millefolium*
4	Backache	*Achilla millefolium, Ferula persica, Juglans regia*
5	Common cold	*Anchusa italica, Echium amoenum, Ziziphora clinopodioides*
6	Constipation	*Malva neglecta, Viola odorata*
7	Diabetes	*Berberis integerrima, Juglans regia*
8	Enteric fever	*Berberis integerrima*
9	Fever	*Viola odorata*
10	Gonalgia	*Juglans regia*
11	Gout	*Ferula persica*
12	Hand schism	*Fumaria asepala*
13	Headache	*Capparis spinosa, Echium amoenum, Fumaria asepala, Heracleum persicum, Stachys lavandulifolia, Viola odorata, Ziziphora clinopodioides*
14	Heart oxygenating	*Ferula persica*
15	Hyperlipidemia	*Berberis integerrima, Grammosciadium platycarpum, Hippophae rhamnoides*
16	Hypertension	*Hippophae rhamnoides, Viola odorata*
17	Increase nausea	*Ziziphora clinopodioides*
No.	Ailment	*Medicinal plants*
18	Infections	*Achilla millefolium, Malva neglecta, Ziziphora clinopodioides*
19	Mange	*Fumaria asepala*
20	Pectoralgia	*Achilla millefolium*
21	Pododynia	*Capparis spinosa, Ferula persica, Juglans regia*
22	Renal calculus	*Capparis spinosa, Stachys lavandulifolia*
23	Sedative	*Echium amoenum, Viola odorata*
24	Sinusitis	*Ferula persica*
25	Stomachache	*Echium amoenum, Ziziphora clinopodioides*
26	Tonic	*Grammosciadium platycarpum, Smyrnium cordifolium*
27	Tremor	*Heracleum persicum*
